# Supplementary material for: Spatiotemporal management of solar, wind and hydropower across continental Europe
Source: Commun Eng. 2024 Jan 5;3:3. doi: 10.1038/s44172-023-00155-3 (PMC10955923; doi:10.1038/s44172-023-00155-3)
Supplement: Supplementary file 2 — Supplementary Information [file 44172_2023_155_MOESM2_ESM.pdf]

Supplementary Information for the article
"Continental Complementarity of Renewable Energy Mixes"

Authors:

Anders Wörman[1,*], Ilias Pechlivanidis[2], Daniela Mewes[1], Joakim Riml[1], Cintia Bertacchi Uvo[3,4]

[1]KTH – Royal Institute of Technology, S-100 44 Stockholm, Sweden
[2]Swedish Meteorological and Hydrological Institute, S-601 76 Norrköping, Sweden
[3]Finish Environmental Institute, FI-00790 Helsinki, Finland
[4]Lund University, S-221 00 Lund, Sweden

[*]To whom correspondence should be addressed; E-mail: worman@kth.se

Content

Supplementary Note 1: Time-series and map details of technical scenarios

Supplementary Note 2: Methods for analysing time-series of mixed renewable power production

Supplementary Note 3: Statistical interpretation of the maximum energy storage in terms of energy security

Supplementary Note 4: Popular illustrations of management mechanisms

Supplementary Note 1: Time-series and map details of technical scenarios

Potential power scenarios are defined based on historical data from the Copernicus ECMWF database, and the monthly consumption pattern is based on that of 19 EU countries from 2009 to 2022. The potential power scenarios are localized to current locations of wind farms (Figure S1) and hydropower plants (Figure S1), whereas solar power is assumed to be uniformly distributed in the analysed domain (Figure S2). The data used and produced in this paper and data reports are available in Zenodo, DOI: 10.5281/zenodo.7750145. Figure S3 shows the historically based power potentials of 642 TWh/y each of solar power (red curve), wind power (green curve) and hydropower (blue curve), aggregated over Europe and parts of the Middle East. The dark blue curve represents a consumption scenario with the same mean power as the monthly pattern produced based on historical fluctuations in electricity consumption data from 19 EU countries. The consumption data used in these analyses are shown in Figure S4.

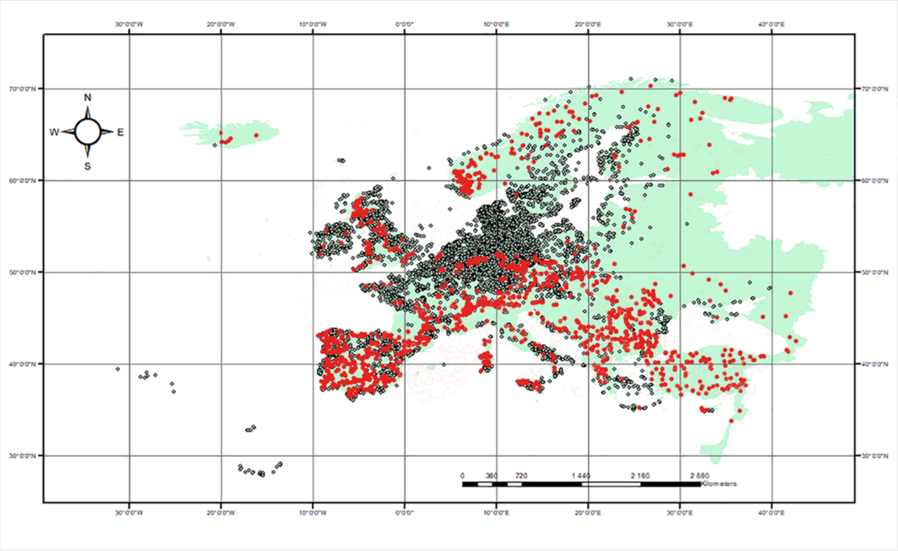

Figure S1 Locations of (1) more than 15,000 onshore wind farms in Europe included in the World Wind Farm database (https://www.thewindpower.net/index.php) (black circles) and (2) 1,377 hydropower stations from the GranD database (Lehner et al., 2011) (red dots). The potential power at these latter stations was estimated from simulated water runoff and generalized to 995 subareas.

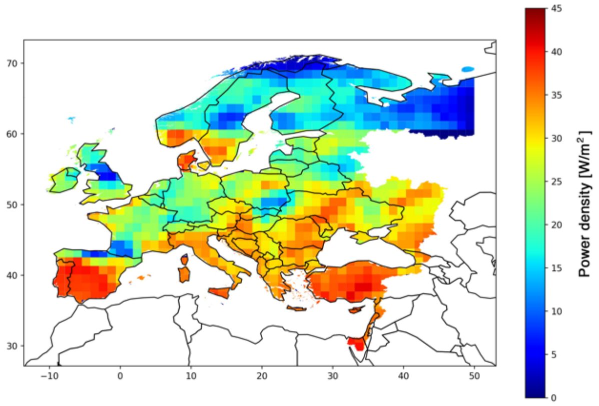

Figure S2 Locations of power time series of PV solar power in W/m^2 on a specific day. The "pixels" are used to average solar, wind and hydropower within 995 subareas, where time series of potential power were determined based on historical data.

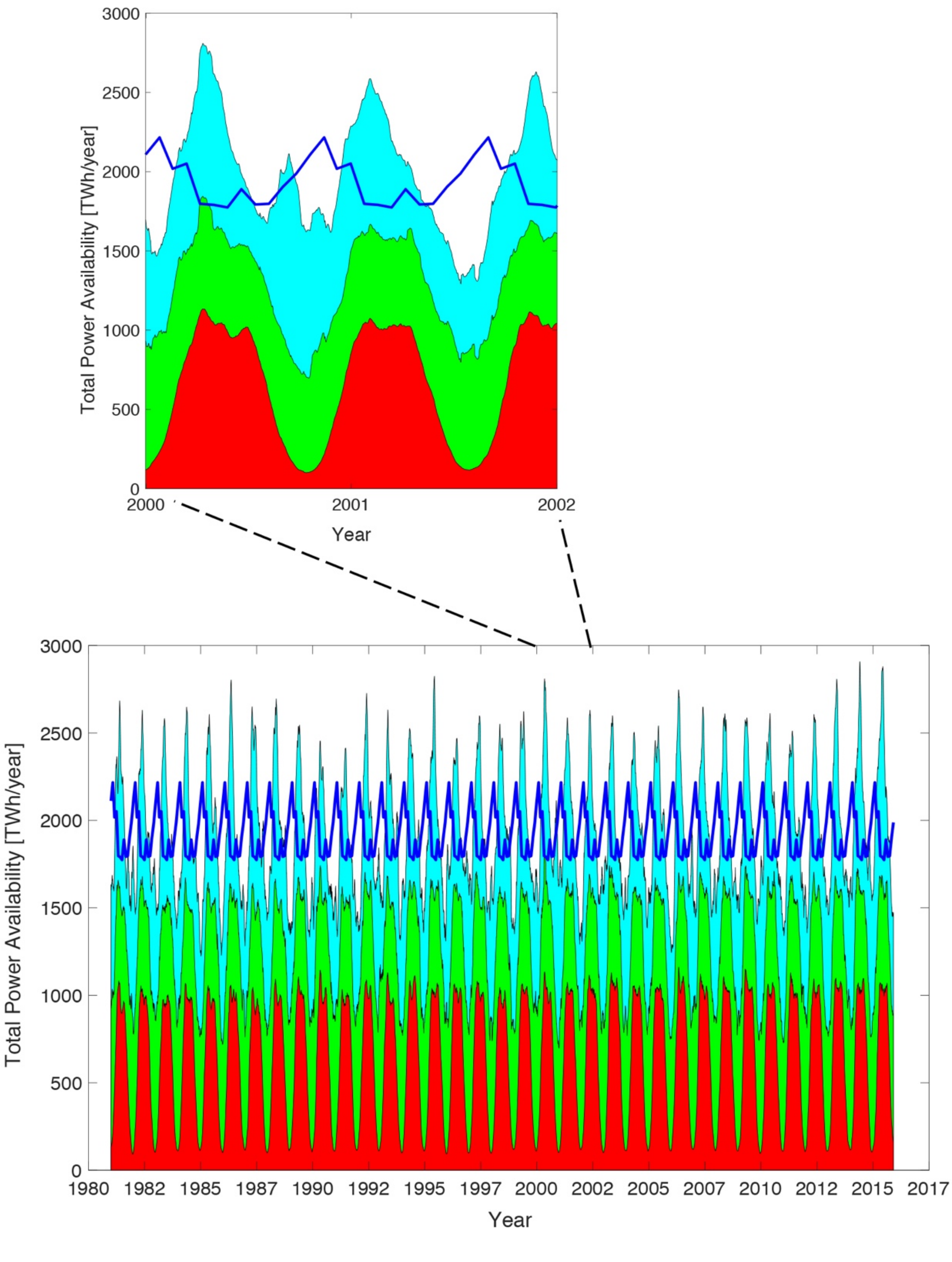

Figure S3 30-day moving average of the daily time series of potential power and electricity consumption for the basic scenario given an equal power potential of 642 TWh/y of solar power (red shaded area), wind power (green shaded area) and hydropower (light blue shaded area). The average is represented as stacked potential power aggregated over the analysed domain; hence, the upper surface of the blue shaded area shows the total potential power variation over time. The dark blue curve represents the daily consumption scenario of 3 x 642 TWh/y, which was derived from the monthly electricity consumption pattern.

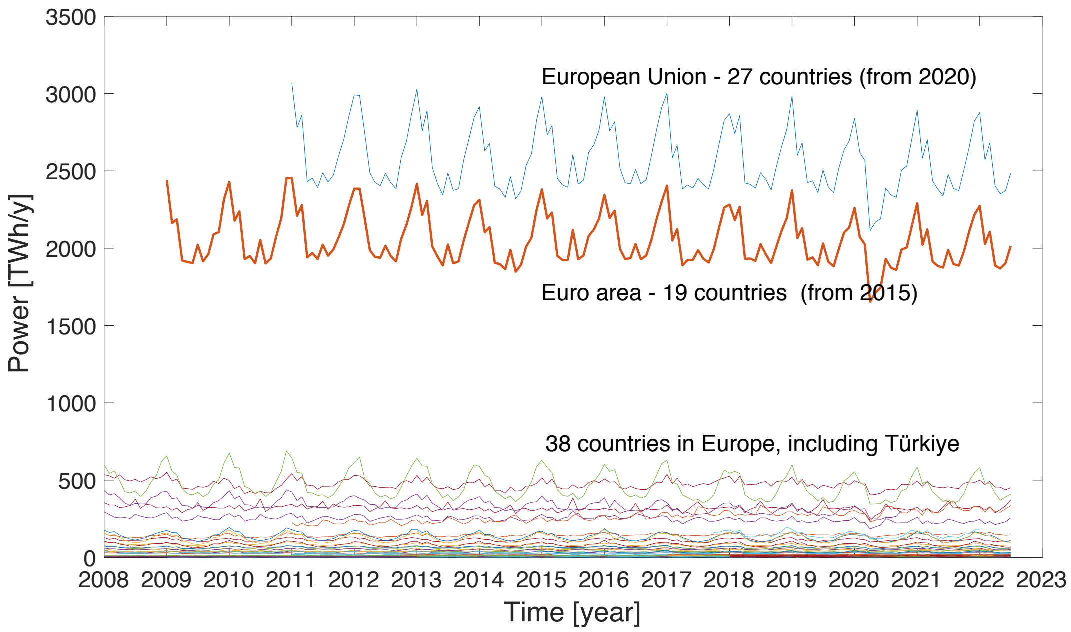

Figure S4 Monthly electricity consumption data for 19 European countries, including Türkiye, for the period 2009–2022 were obtained from the Eurostat database "Energy statistics – quantities", as part of the theme "Environment and Energy". The monthly data were aggregated for all countries and generalized with a daily resolution for the same period (red curve). To obtain a representative pattern of electricity consumption, the monthly data from the 13 years were averaged and then aggregated and generalized on a daily basis for the period from 1 January 1979 to 31 December 2015 (dark blue curve of Figure S3).

Supplementary Note 2: Methods for analysing time-series of mixed renewable power production

S2.1. Power spectra as an indication of the variance in the power potential

A basic energy conservation representing the required energy storage can be expressed as $\partial E/\partial t = P - P_C - P_S = P_\delta$, where E = stored energy (J), P = actual power production (W), P_S = spilled power (not produced while available) and otherwise exported energy (not considered consumed energy), P_C = power consumption (W), and P_δ = power production deviation resulting in a time rate of change in energy storage demand. This analysis assumes that the required energy storage, E, is provided, and spillage arises when the installed power capacity P is larger than the minimum level P_{min} required to match the average consumption in the long term. The energy balance can be equated as:

$$\frac{\partial E}{\partial t} = \alpha P_{min} - P_C - P_S \qquad \text{(A1)}$$

where the excess power coefficient $\alpha = P/P_{min}$ is derived from the expression $E[(\alpha - 1)\, P_{min}] = E[P_S]$, where $E[\ldots]$ is an operator defining the long-term expected value. While zero excess power capacity $(\alpha = 1)$ is the focus of this paper, excess capacity can be useful for reducing the energy storage demand. In particular, increasing the installed power capacity can reduce

the energy storage demand, which is similar to the effects of complementarity and spatiotemporal coordination between renewable energy sources (Supplementary Note 4).

For simple time series, such as single harmonic variations of P_δ, one can estimate the maximum required energy storage as $E_{max} = \sqrt{2}\ Std(P_\delta)\ T/\pi$, where the period *T = 1/f (s)* and *f* = frequency (s^{-1}). However, for composite time series, in this case one reflecting a variety of climate signals, the required energy storage *E* is estimated using a spectral transform of basic energy conservation (Wörman et al., 2020):

$$Std(E) = \sqrt{2\int_{f_1}^{f_2} S(E(f))df} = \sqrt{2\int_{f_1}^{f_2} \frac{S(P_\delta)}{(2\,\pi\, f)^2} df} \qquad \text{(A2)}$$

where *S(E)* = power spectral density of *E*, $S(P_\delta)$ = power spectral density of P_δ, f_1 = the lower frequency (s^{-1}), and f_2 = the higher frequency (s^{-1}). Hence, by using power spectral transforms, Equation A2 allows the standard deviation of the stored energy to be estimated rather than its maximum value. In addition, the variance in the production signal itself can be expressed as:

$$Var(P) = 2\int_{f_1}^{f_2} S(P)df \qquad \text{(A3)}$$

The relationship between the maximum energy storage demand, E_{max}, and the standard deviation in the energy storage demand as expressed by Eqn. (A2) is further detailed in section (A3). For periodic and therefore autocorrelated time series, it is relevant to assess this relationship while accounting for both the regulatory period under consideration and the frequency with which the energy storage demand is exceeded. For a multiannual regulatory horizon, one can approximate $E_{max} = C\ Std(E)$ if *Std(E)* is assessed over all frequencies of *S(P),* where *C* is a constant in the range of 1 to 1.5 corresponding to the regulatory time horizon spanning from 3 to 5 years (See Supplementary Note 3). This proportionality ignores the energy storage demand arising over decadal or longer-term climate variations.

S2.2 Covariance spectra of spatiotemporal coordinated power time-series

When different power stations from different energy sources are considered, the power spectrum density of a sum of time series can generally be expressed as $S(\sum_{i=1}^{N} P_i) = \sum_{i=1}^{N} S(P_i) + \sum_{i\neq j}^{N} \mathrm{Re}\{S(P_i;P_j)\}$, where $S(P_i;P_j)$ is the cross-spectrum (or cross-covariance spectrum) between power capacities P_i and P_j. This expression reflects the cross-correlation between the power production at all power stations and assumes that there are no electric transmission limits on power production. Spatiotemporal coordination can also consider only a subset of power station pairs for which this cross-covariance is calculated, whereas the remaining station pairs are disregarded in this situation. Hence, a general expression of the power spectral density of the power production considering spatiotemporal coordination is

$$S(\sum_{i=1}^{N} P_i) = \sum_{i=1}^{N} S(P_i) + \sum_{i\neq j}^{N} \mathrm{Re}\{S(P_i;P_j)\}\Big|_{F(i,j)=1} + \sum_{i\neq j}^{N} \sqrt{S(P_i)S(P_j)}\Big|_{F(i,j)=0} \qquad \text{(A4)}$$

where Re denotes the real part of the spectra and $F(i,j)$ is a conditional, bimodal function that takes the value 1 when the cross-correlation between two power stations is considered or the value 0 when the two power stations are operating independently. In this study, we considered station pairs at distances $r_{i,j}$ within a coordination distance R as subjects from spatiotemporal coordination; hence, for all pairs F(i,j) = H(R - $r_{i,j}$), where H = the Heaviside function. Equation (A4) reflects those independent operations (0 < R - $r_{i,j}$) maximize the variance in power production and thus maximize the reserve power capacity or energy storage demand.

S2.3 Covariance spectra of aggregated solar power, wind power and hydropower

The complementary characteristics between the renewable energy sources can be assessed using a spectral transform of the basic energy equation without spillage: $\partial E/\partial t = P - P_C$. We separate the potential power of the renewable energy mixture into its three sources: $P = P_H + P_S + P_W$, where the power production of the individual sources are expressed as $P_H = \sum_{i=1}^{N} P_i\big|_H$, $P_S = \sum_{i=1}^{N} P_i\big|_S$ and $P_W = \sum_{i=1}^{N} P_i\big|_W$. Furthermore, if P_C is the aggregated electric consumption, the power spectral density of the power deviation $P - P_C$ is expressed as

$$S(P - P_C) = S(P_H) + S(P_S) + S(P_W) + S(P_C) + 2Re\{S(P_H, P_S)\} + 2Re\{S(P_H, P_W)\} + 2Re\{S(P_S, P_W)\} + 2Re\{S(P_H, -P_C)\} + 2Re\{S(P_S, -P_C)\} + 2Re\{S(P_W, -P_C)\} \tag{A5}$$

To emphasize the covariance between potential production and consumption, Eqn. (A5) can also be rewritten as

$$S(P - P_C) = S(P_H + P_S + P_W) + S(P_C) - 2\,Re\{S((P_H + P_S + P_W), P_C)\} \tag{A6}$$

S2.4 Numerical implementation of spectral analyses

Before power-time series are spectral transformed, the series are detrended and filtered using Hamming windowing on the entire time-series. The spectral analysis is then conducted using Welch's method as implemented in the CPSD function of MATLAB®. Frequencies are distributed over 256 unisized frequency steps from zero to half the data frequency (0.05 days^{-1}), which leads to a nonuniform distribution of period steps. This procedure ensures that the energy storage demand identified from the 35-year data records does not depend on possible record trends but on fluctuations significantly shorter than 35 years. Hence, the longest period considered in the spectral procedure is 14 years. Since Eq. (A4) accounted for 13.6 million cross-spectra, the computational efforts must be reduced by averaging the daily power production time series using 10-day steps. The monthly pattern of electricity consumption is also derived using a 10-day window.

Supplementary Note 3: Statistical interpretation of the maximum energy storage in terms of energy security

The standard deviation of the instantaneous storage demand was assessed by spectral decomposition. This assessment is motivated by the fact that hydroclimatic time-series are generally combinations of periodic and apparently random components, which is reflected in the functional trends of the power spectra and superimposed (random) noise (Figure 3a). Consequently, two essential statistical aspects affect the energy storage demand E_{SD} and the degree of energy security reflected in the storage demand concept:

1) The maximum energy storage demand depends on the selected *exceedance probability* defined by the partly random distribution of the instantaneous energy storage demand, and
2) The energy storage demand depends on the *regulatory periods* considered when balancing the power fluctuations, i.e., daily, monthly, seasonally or multiannually.

As an example, Eqns. (A2) and (A3) imply that the variance of the distributions in E or P can be limited to the bounds of the considered periodic variation frequencies f_1 and f_2. Excluding some frequency intervals decreases the variances and event (e.g. energy drought or storage demand exceedance) probabilities accordingly. This suggests that the reliability of an energy storage demand always being satisfied depends on both the selected regulatory periods (or time horizon) and the random storage variations. For a single sinusoidal variation in the energy storage over time, the maximum (amplitude) energy storage demand is given by

$$E_{max} = \sqrt{2}\ Std(E) \qquad \text{(A7)}$$

For an unbounded random distribution, such as Gaussian or Weibull, the storage demand is linearly proportional to the standard deviation in the storage but also depends on the exceedance probability of the considered maximum bound:

$$E_{max} = a(p)\ Std(E) \qquad \text{(A8)}$$

where *a(p)* is a proportionality coefficient that depends on the probability *p* that V_i exceeds certain bounds (exceedance probability) of the distribution of V_i. Eqn. (A2) implies that the energy storage demand can be limited to the longest considered period $T_1 = 1/f_1$, in the form $E_{max} = a(p)\ Std(E)|_{T1}$, where the period-limited standard deviation $[Std(E)|_{T1}] = b(T_1)\ [Std(E)]$, where $b(T_1)$ = a coefficient less than 1 that is dependent on the limiting period T_1. Thus, the maximum energy storage can be expressed as a function of the two coefficients *a(p)* and $b(T_1)$ in the form

$$E_{max} = a(p)\ b(T_1)\ Std(E) \qquad \text{(A9)}$$

Coefficients a(p) and $b(T_1)$ represent the time-series variability, where *a(p)* considers the randomness expressed by the probability density function and $b(T_1)$ considers the periodic nature of the same variability. The arguments in sections A3.1 – A3.4 suggest that the product a x b = 1.47 (a ≈ 3.265 and b = 0.45) when a regulatory period of 5 years is applied, and a x b = 1 for a regulatory period of three years (a = 3.09 and b = 0.35).

S3.1 The maximum energy storage demand due to the exceedance probability

While the *return period* is a term normally applied for independent data, which is strictly not applicable to the autocorrelated, periodic time series in this paper, defining the exceedance probability in such time series as a qualitative measure of the return period is generally possible. For unbounded distributions, such as Gaussian and Weibull distributions, we can use the cumulative distribution function of E_i to define the coefficient $a(p)^2 = z_p^2$, where z_p is the upper (positive) p-quantile associated with the exceedance probability *p*. For the standard normal Gaussian distribution, we express the exceedance probability as $p\left(\frac{(E_i-\mu_{E,i})}{\sigma_{E,i}} > z_p\right) = \left[1 - erf\left(z_p/\sqrt{2}\right)\right]$, where $\left(E_i - \mu_{E,i}\right)/\sigma_{E,i} \in N(0,1)$ and *erf* denotes the error function. For example, the first millile of the daily data (p = 0.001 $days^{-1}$, or a return period of 1/p = 1,000 days or three years for the exceedance event) is z_p = 3.09; hence, $E_{max} = 3.09\ Std(E)$. If the return period for the exceedance of the energy storage demand is taken as five years (p = 5.473 10^{-4} $days^{-1}$), we have $E_{max} = 3.265\ Std(E)$. For a three-year return period, a = 3.09, and for a five-year return period, a = 3.265.

For the Weibull distribution, the exceedance probability $p\left(E_i/\lambda > z_p\right) = \exp\left(-\left(\frac{E_i}{\lambda}\right)^k\right)$, where k is a shape factor and λ is the scale. A numerical example of the energy storage distribution is provided in Supplementary Note 3: S3.3. In this example, the shape factor was found to be k = 2.2. Hence, the exceedance probabilities of 0.001 $days^{-1}$ and 5.473 10^{-4} $days^{-1}$ correspond to E_i/λ values of 2.40 and 2.50, respectively. Furthermore, when the variance of the Weibull distribution equals $(\lambda/k)^{2,}$ we can estimate the coefficient a = 5.25 and 5.50 for the two probabilities.

S3.2 Energy storage demand due to selection of an upper the regulatory period

Spectrally decomposing the energy storage demand allows us to separate the demands on the dominating periods of variability in the energy access and to consider only relevant period bands when regulating power fluctuations. Specifically, the assessment of the energy storage demand according to Eq. (A2) can be ended at the lowest frequency f_1 considered for the regulation time horizon, i.e., the longest regulatory period $T_1 = 1/f_1$. Figures S5 and S6 show the dependence of the energy storage demand on the coordination distance as well as on the longest period considered for spatiotemporal coordination. The relative importance of spatiotemporal coordination on the percentage VESG is fairly equal across the band of considered maximum periods, $1/f_1$ (Table S1). Notably, the longest regulatory period T_{max} that can be considered in the spectral analysis is half of the length of the considered time series, which was T_{max} = 35/2 = 17.5 years here. To meet conditions for Welch's spectral method in MATLAB® (Supplementary Note 2: S2.4), this period was further reduced to approximately 14 years (shown as > 10 years in Table S1 and Figures S5 and S6). Figure S5 illustrates that the energy storage demand accounting for the longest lasting power fluctuations is related to the 5-year regulatory period by $E_{SD,14} = 2.2\ E_{SD,5}$ when *R = 0*, i.e., the coefficient b = 0.45 in Eqn. (A9). For the three-year regulatory period, b = 0.35. While the magnitude of power deviations decay as a function of the regulatory period, the energy storage demand increases by a factor of 2 as T goes from 5 to 17 years (see Figures S5 and S6). This, is due to the fact that the energy storage demand depends on the product of the magnitude in power deficit and the duration of the deficit.

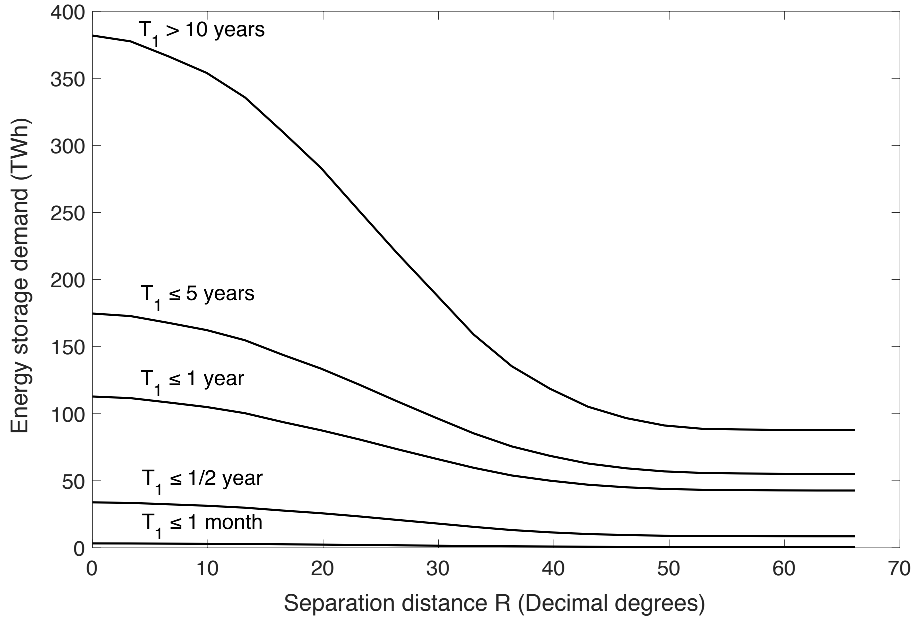

Figure S5 Energy storage demand decomposed on different regulatory periods for regulating the power balance in the scenario with 1:1:1 shares of solar-wind-hydro power. Here, the demand was set as a constant to demonstrate the influence of periods on only the potential power production.

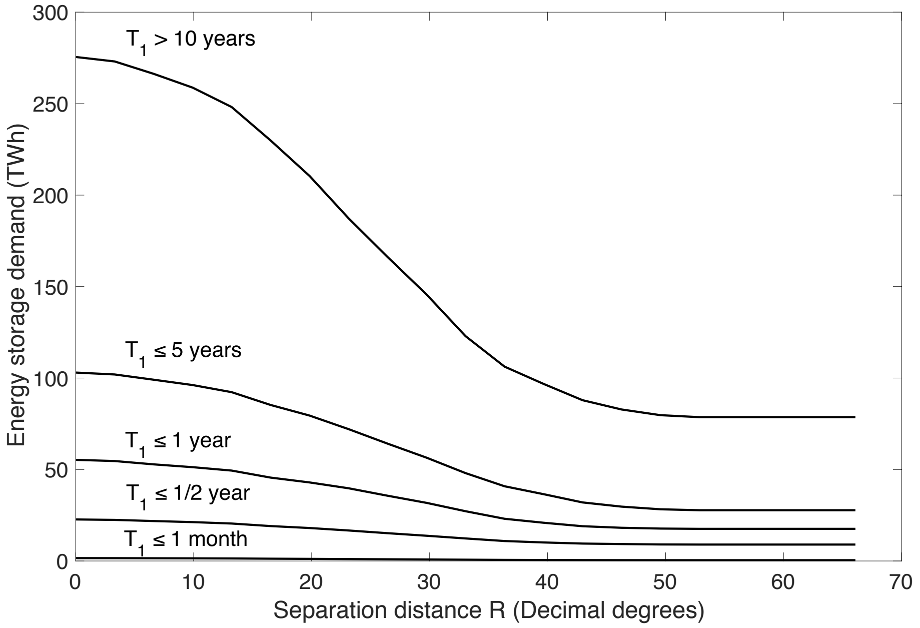

Figure S6 Energy storage demand decomposed on different regulatory periods for regulating the power balance in the scenario with 0:0:1 (only hydro) shares of solar-wind-hydro power. Here, the demand was set as a constant to demonstrate the influence of periods on only the potential power production.

Table S1: Summary of E_{SD} and VESG assessed from Figures S5 and S6.

Regulatory period	E_{SD}(R=0) [TWh]	E_{SD}(R=∞) [TWh]	VESG	% VESG
**Solar-wind-hydro 1:1:1 scenario (3 x 642 TWh/y)**				
**> 10 years**	381.9	87.7	294.2	77.0
**< 5 years**	174.7	55.1	119.6	68.5
**< 1 year**	112.8	42.7	70.1	62.1
**< 0.5 year**	33.9	8.57	25.3	74.6
**< 1 month**	3.27	0.63	2.64	80.7
**Only hydro (642 TWh/y)**				
**> 10 years**	275.5	78.6	196.9	71.5
**< 5 years**	103.0	27.7	75.3	73.1
**< 1 year**	55.3	17.6	37.7	68.2
**< 0.5 year**	22.7	9.0	13.7	60.3
**< 1 month**	1.56	0.43	1.13	72.4

S3.3 Numerical example of the distribution of storage demand

The spectral approach expresses neither the instantaneous energy storage demand nor the corresponding probability density function (PDF) of storage demand. We thus derived a numerical solution to Eqn. (A1) in order to express these variables for the scenario with 1:1:1 shares of solar-wind-hydropower (Figure S3). Figure S7 shows the PDF of the daily data of the aggregated power in this scenario with fitted distribution functions, which demonstrates that the available renewable power can vary by a factor of 4 even when aggregated over the entire European continent. Thus, certain days clearly exhibit very low solar power, wind power, and hydropower and thus require a significant amount of stored energy, such as in hydropower reservoirs. The probability density has a significant degree of uncertainty at the extremes, which prevents the return periods for energy droughts from being reliably estimated. Nevertheless, Figure S8 shows the associated development of the energy storage demand over time, and Figure S9 shows the PDF of the individual daily storage values with two fitted distribution functions, Weibull and Gauss. The standard deviation of the instantaneous energy storage demand is 139.9 TWh for the 35-year-long time series but this value is highly influenced by the decadal-long variability. In the period from 1 January 1992 to 31 December 2000, the standard deviation is only 66.7 TWh.

The significant decadal-long variability in energy storage reflects the long-lasting lows in energy access in the climate system. The importance of duration of low energy availability (energy droughts) can be demonstrated by evaluating the return period of the annual minimum event of the energy droughts. The power distributions shown in Figure S7 is transformed for various moving average window from 1 day to 15 years, while the corresponding return periods of the annual minimum (worst) energy droughts are shown in Figure S10. The energy storage demand corresponding to the five-year return period is shown in Table S2 for the range of moving average windows. These numerical results confirm the findings from the spectral analysis regarding both the level of energy storage demand and the relative importance of period or duration of the energy drought events for the energy storage demand.

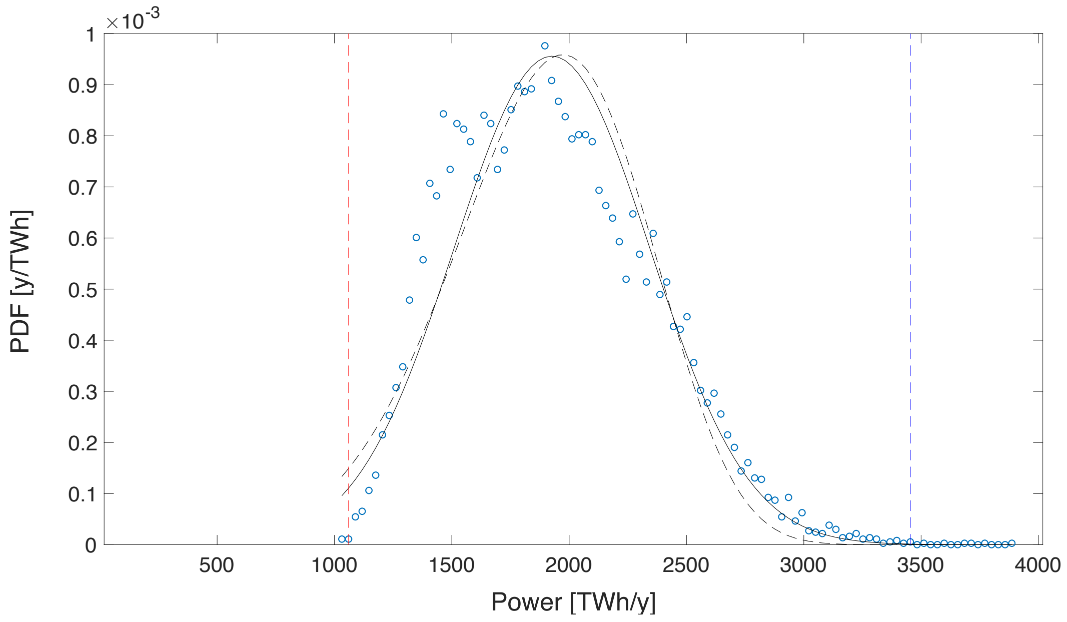

Figure S7 PDF of the aggregated daily power for the technical scenario with 1:1:1 shares of solar, wind and hydropower. The solid black curve represents the Gaussian distribution (with mean = 1,927 and standard deviation = 417), and the dashed curve is the Weibull distribution (k = 5.25 and λ = 2,054). The vertical dashed lines correspond to the five-year return periods using the Gaussian distribution to determine the aggregated power that is not exceeded (red line) and is exceeded (blue line). Given the significant variance in the renewable energy distribution even over large areas, spatiotemporal coordination of production can reduce the physical energy storage needs and overcome energy droughts.

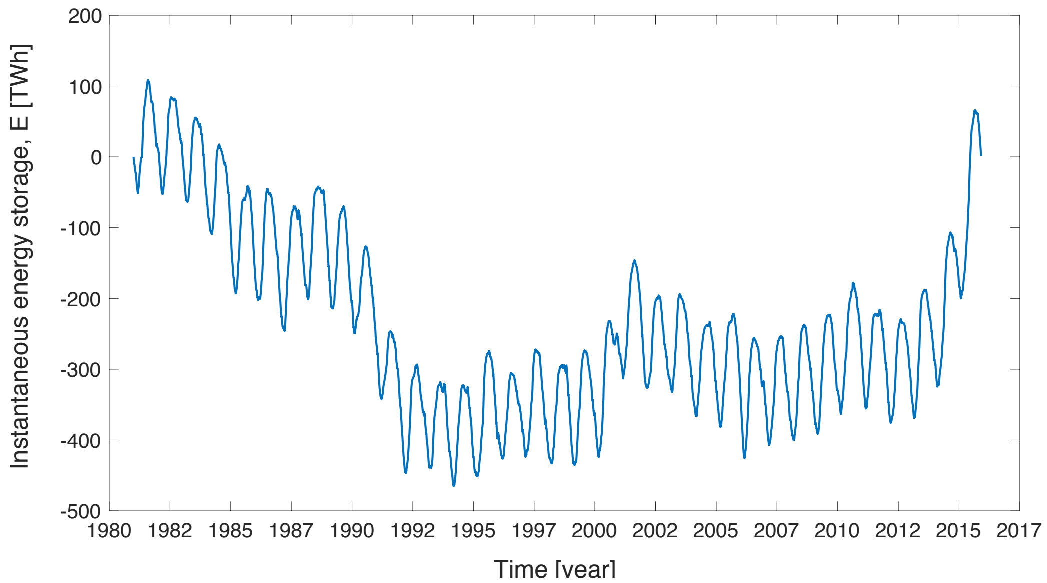

Figure S8 Numerical evaluation of the instantaneous energy storage for the power system with 1:1:1 shares of solar, wind and hydropower as an aggregated time series across Europe. The solution is based on a finite difference integration of Eq. (A1) using both the potential power time-series and the consumption scenario described in the Methods and shown graphically in Supplementary Note 1. The negative storage values are caused by the zero initial value.

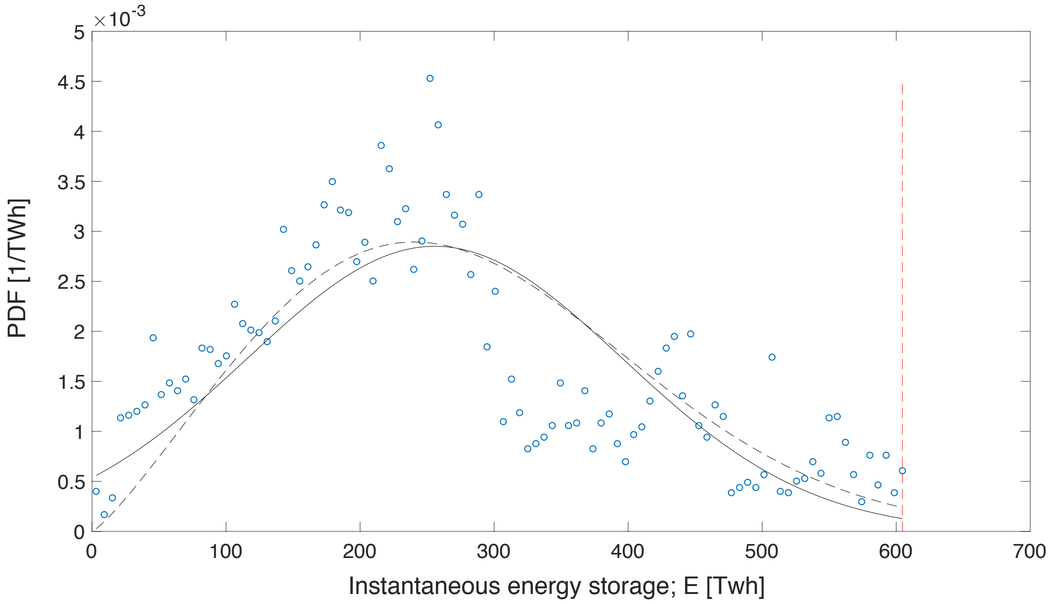

Figure S9 PDF of the aggregated energy storage demand for the technical scenario with 1:1:1 shares of solar, wind and hydropower. The solid black curve represents the Gaussian distribution (with mean = 255.8 and standard deviation = 139.9), and the dashed curve is the Weibull distribution (k = 2.2 and λ = 316.6). The red vertical dashed line corresponds to the five-year return period for exceeding storage demand.

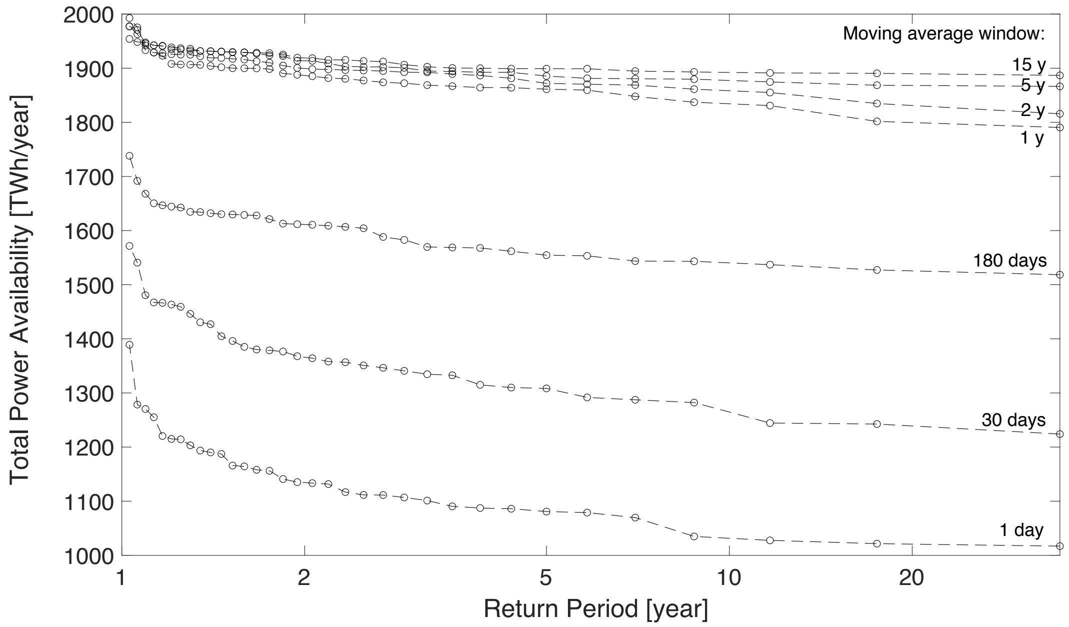

Figure S10 Return period for annual energy drought events for which the available power falls below a certain value at different moving average windows. The diagram is derived for the technical scenario with 1:1:1 shares of solar, wind and hydropower.

Table S2 Storage demand arising at energy drought events with a 5-years return period. The deficit in column 2 is obtained as the difference between the mean power (1,927 TWh/y) and the power during the drought given a specific moving average window (values are obtained from Figure S10).

**Moving average window**	**Power deficit at 5-year return period [TWh/y]**	**Energy storage demand [TWh]**
1 day	1927 – 1081 = 846	846 x 1/365 = 2.30
30 days	1927 – 1308 = 619	619 x 30/365 = 59.9
180 days	1927 – 1554 = 373	373 x 180/365 = 184
1 year	1927 – 1861 = 66	66 x 365/365 = 66.0
2 years	1927 – 1872 = 55	55 x 2 = 110
5 years	1927 – 1886 = 41	41 x 5 = 205
15 years	1927 – 1899 = 28	28 x 15 = 420

Supplementary Note 4: Popular illustrations of management mechanisms

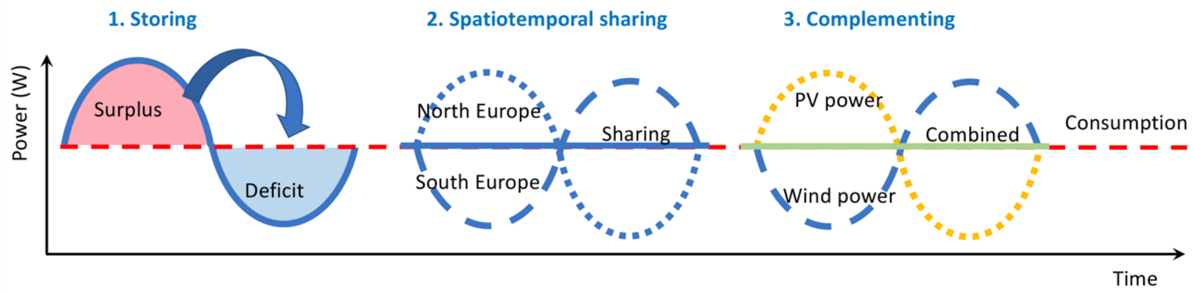

Figure S11 Three major mechanisms exist for matching the variable availability of solar-wind-hydropower to electricity consumption: 1) storing energy primarily in hydropower reservoirs, 2) spatiotemporal sharing or managing the electricity system throughout Europe or 3) providing complementary production capacity from the energy sources to appropriately reflect the hydroclimatic variabilities. Since renewable energy potential varies across Europe, there are important connections between spatiotemporal sharing and complementary production.

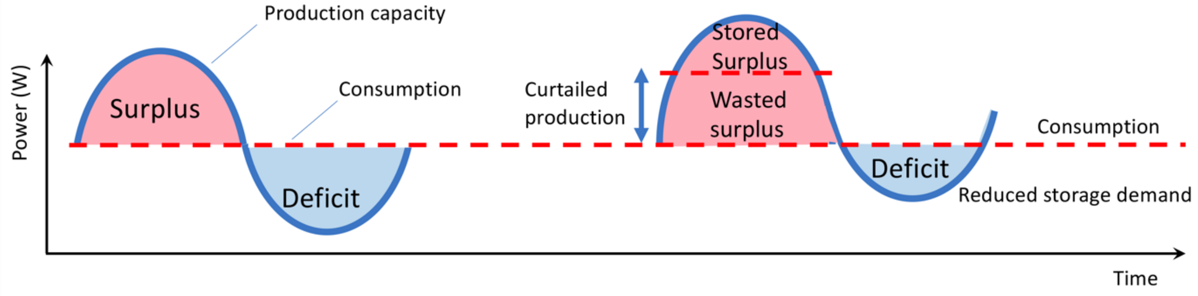

Figure S12 Increasing the production capacity increases the potential of energy being dispatched outside the electricity consumption domain but simultaneously reduces the energy storage demand. Introducing additional production capacity can be seen as a fourth mechanism for matching the variable availability of solar-wind-hydropower to the electricity consumption.

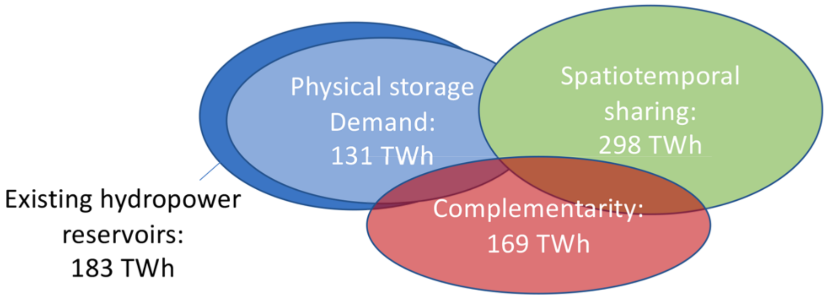

Figure S13 Storage shares for the scenario with 2:4:1 shares of solar-wind-hydro power. In this scenario, the power sources provide 4,494 TWh/y over the investigated domain, and all three matching mechanisms are required to fulfil the energy storage demand. The utilized energy storage within existing hydropower is 131 TWh for the 2:4:1 scenario, whereas the available hydropower energy storage of 183 TWh.
